# Mindset switching increases the use of '*want-based*' over '*should-based*' behaviors

**DOI:** 10.1371/journal.pone.0196269

**Published:** 2018-04-30

**Authors:** Jin Yan, Nan-Nan Zhang, Dai-Xuan Xu

**Affiliations:** 1 School of Management, Zhejiang University, Hangzhou, China; 2 Faculty of Economics and Business, HRM & OB, University of Groningen, Groningen, The Netherlands; 3 Antai College of Economics and Management, Shanghai Jiao Tong University, Shanghai, China; Technion Israel Institute of Technology, ISRAEL

## Abstract

This study examines the consequences of mindset switching on behavioral choices in *want*/*should* conflicts. Building on the insights of the ego depletion literature, we propose that mindset switching depletes individuals’ self-control resources and therefore prompts the choice of *want* behavior, which provides immediate pleasure, over *should* behavior, which provides long-term utility. Four laboratory experiments with university students that stimulated individuals to switch mindsets were conducted to test our hypotheses. Experiment 1 demonstrated that switching between individualist and collectivist mindsets increased the subjects’ tendency to prefer popular magazines over scientific journals. Experiment 2 replicated the results by testing the relationship between an abstract/concrete mindset-switching task and *want*/*should* online behavioral choices. The mediating effect of ego depletion was also supported. Experiment 3 retested the main effect of language-switching on reading choices, and the mediating effect of ego-depletion. Experiment 3 also tested the moderating effect of the Need for Cognition, and eliminated the alternative explanation of cognitive fatigue. In Experiment 4, actual food choices were used as the direct measure of *want*/*should* behaviors to test the robustness of our findings. The results consistently supported our hypotheses that mindset switching has significant effects on behavioral choices in terms of overindulgence, such as increasing *want* behavior and thus foregoing *should* behavior.

## Introduction

Mindset switching, switching back and forth between different mental states, is a common practice in daily work for most workers. Often, managers have difficulty concentrating on a single task. Schulte found that typical managers were interrupted or switched tasks, on average, about every three minutes [1]. The Internet provides great distractions for managers and students alike. Ravizza, Uitvlugt, and Fenn found that university students spent a median of 37 minutes per class surfing the Internet for nonacademic purposes [2]. In the psychology literature, scholars use the term *mindset switching* [3], also *task switching* and *shifting* [4, 5], to describe the behavior of switching between mental tasks.

Numerous studies have explored the effects of mindset switching on subsequent behavior. For instance, many studies demonstrate the negative effects of task switching, such as impeding cognitive performance [6] and increasing errors [7]. However, a recent study conversely shows the positive effects of task switching on creativity [8]. These mixed findings call for further exploration of the mechanism through which mindset switching affects behaviors.

This study is designed to answer the questions of why and how mindset switching affects individuals’ preferences when they face a variety of choices. The findings that individuals including managers and students frequently switch tasks suggest that they generally face competing task choices. When performing a goal-oriented task, besides the efforts of self-control, people often feel internal motivational conflict to choose among a variety choices [9]. Following a thorough search, we find a gap in the literatures on the effects of mindset switching on preferences, thus we set out to explore these effects.

This study aims to understand how mindset switching influences individuals’ choice when faced with *want* and *should* options. *Want* options provide individuals with immediate gratification, while *should* options offer delayed gratification and serve long-term objectives. Using the ego depletion theory [10, 11] and established measures of *want*/*should* behavior [9, 12], we propose that mindset switching depletes our self-control resources, leading us to choose *want* behavior over *should* behavior. In other words, we tend to overlook long-term goals and choose immediate rewards after mindset switching.

### Mindset switching

The terms *shifting* and *mindset switching* refer to switching back and forth between mental tasks, yet the terms have slight differences. The term *shifting*, also referred as *attention switching* or *task switching*, describes the process of switching between multiple cognitive operations [4, 7]. Task shifting involves the disengagement of a previously performed mental operation and the subsequent active engagement in a new operation. The ability to shift between tasks is considered a basic aspect of executive functions and has helped researchers to better understand the failures of cognitive control [6, 13]. Executive function helps individuals to perform various primary tasks such as to plan and organize, attend to a speaker, follow a process, manage time, and switch focus when necessary. In a series of experiments, Miyake required the participants to switch back and forth between opposite cognitive operations, such as in a plus-minus task, a number-letter task, and the local-global task to manipulate shifting [4]. However, shifting describes the switching of an individual’s cognitive operations, but not the switching of general metal states.

The term *mindset switching* reflects a more abstract definition of the switching back and forth between mental modes. Hamilton et al. [3] distinguished the concept of mindset from cognitive operations. They argued that a mindset is more general than a mental operation, which involves a single task completion. According to Hamilton et al., an individual’s mindset facilitates mental states that are not specific for one task but represent a global mental readiness to respond to multiple tasks. A mindset is the particular way in which an individual reacts to stimuli and processes social information [14], such as *regulatory focus* [15], *abstract/concrete mindsets* [16], and *culture-specific mindsets* [17]. Mindsets are sticky and mutually exclusive [3]. Sticky means that they can influence an individual’s behavior across different tasks [14]. Mindsets remain active beyond initial tasks, thereby influencing subsequent tasks. An individual must overcome the negative thinking inertia, when switching to another mindset. Moreover, mindsets are mutually exclusive, and it is difficult to simultaneously adopt more than one mindset. When one mindset is active, activating another mindset requires switching away from the current one. Thus, we can summarize that mindset, a state of global mental readiness to react, is sticky and mutually exclusive.

Correspondingly, mindset switching means to shift from one mindset to another. Situational cues, like the demands of a particular task, can prompt individuals to switch from one to another mindset. For example, some chronic mindsets, such as Eastern or Western cultural mindsets [17] or chronic regulatory focus formed by living experience[15], can be temporarily switched by situational priming (e.g., answering questions in Eastern or Western mindset). When experimenters manipulate their study subjects to switch their mindsets, they present situations that require subjects to switch back and forth between two opposite mindsets, such as Eastern to Western, promotion to prevention, or abstract to concrete mindsets [3].

The discussion above can be summarized by the notion that *task shifting* and *mindset switching* are related but different theoretical terms. Task shifting refers to the shifting of specific cognitive operations, whereas mindset switching refers to a changing of global mental readiness. In this study, we use the term *mindset switching* to describe the mental transitions that managers faced. Managers often do work that requires higher-level thinking such as writing strategic plans and reports, creating marketing or project presentations, supervising employees, making tactical and strategic decisions and supervising personnel. Let us imagine that when a manager write a sales report, he/she spends some time, let’s say 10 minutes, collecting his/her thoughts and writing an outline. And then, the manager begins writing for about 10 minutes before get interrupted by a phone call. After dealing with the caller, the manager returns to writing again. But his/her mind has been pulled away from the report, and another 5 to 10 minutes are required to collect his/her thoughts and return to the writing process again. In the above situation, the manager has switched his/her attention from report writing to the phone caller’s request or information on an entirely different matter, but has also disengaged from the mental mindset of report writing. For this reason, we believe that the term *mindset switching* better explains the manager’s behavior than *task shifting*. Therefore, we use the term *mindset switching* in this study referring to instances when someone is interrupted in a task and must return to it.

### *Want*/*Should* conflict

We use the *want*/*should* behavior to test behavioral choices. An individual faces a variety of choices when making decisions and in doing so, must balance his/her unique motivations. Bazerman and Tenbrunsel argue that when people make decisions, they balance their desires (i.e., motivation) between instantaneous pleasure and long-term utility [9]. However, it is often difficult for individuals to resist the choice that brings instantaneous pleasure. For instance, if someone is offered a slice of chocolate cake or a carrot at a party, most people would find it difficult to take the carrot, far more healthy than sugary cake. The urge to taste rich dark chocolate would outweigh the vitamins to be gained from the crisp orange carrot and so they would choose the “dead” calories of the delicious cake. In other words, the desire for immediate gratification would outweigh the far more rational choice of long-term health. We are often torn between two competing motivations and balancing them requires self-control. Thus, the *want*/*should* behavior model was developed to describe internal motivation conflicts [9, 12].

Psychologists have developed several “multi-selves” models to explain internal motivational conflicts. For example, Thaler [18] highlights the conflict using the *planner* and *doer* mindset when individuals consider making savings plans. Schelling [19] proposed that we experience conflict between what is best for long-term utility and what brings us immediate pleasure. Higgins [20] proposed the self-discrepancy model whereby discrepancies exist between “self-state representations”, namely the actual (“want to”) self and the ideal (or “ought to”) self. Much research addresses this topic; the first empirical study, by Bazerman, Tenbrunsel, and Wade-Benzoni [9] provided empirical evidence that individuals face internal conflicts due to tradeoffs between their *want* and *should* selves.

The *want*/*should* conflict arises when individuals face multiple choices. For example, an overweight individual might struggle between ordering a high-calorie yet delicious cheese pizza or a healthy salad with vinegar dressing. On the weekends, a stressed-out manager might deliberate whether to relax and watch her favorite television show or go for a half-hour jog. What they *want to do* conflicts with what they know they *should* do. Milkman et al. [12] summarized the model of the *want*/*should* self and corresponding behavioral choices. First, the instantaneous payoff (e.g., pleasure) obtained from *want* (hedonic) choices is greater than the payoff obtained from *should* (utilitarian) choices, which generally bring delayed satisfaction. Second, the sum of the utilities obtained from *should* choices is greater than that obtained from *want* choices throughout the whole period. As illustrated by Bazerman et al. [9], the *want* self is emotional and impulsive, whereas the *should* self is rational and pragmatic. Researchers have used food choices to measure *want*/*should* behavior in empirical research [21, 22].Essentially, individuals can achieve a healthier state if they order *should* foods (i.e., a lean salad) instead of *want* foods (a pizza). However, to achieve a healthy state, eaters must exert effort to resist instantaneous pleasure, their *wants*, and pursue the long-term utility that they know they *should*.

Inzlicht, Schmeichel, and Macrae [23] also address similar concepts of *want-to* and *have-to* goals. They argue that individuals must balance the motivational needs of cognitive leisure (*want-to* goals) and cognitive labor (*have-to* goals) when engaging in goal-oriented mental processes. *Have-to* goals involve a sense of duty or obligation to individuals, whereas *want-to* goals are personally enjoyable and meaningful. As such, *want-to* tasks are much easier to perform than *have-to* tasks. *Have-to* and *want-to* goal-oriented behaviors are similar to our study’s *should* and *want* behaviors, respectively. A goal-oriented action suggests that the individual monitored his/her *have-to* goals and simultaneously inhibited his/her *want-to* goals.

While Inzlicht et al. [23] did not perform empirical manipulations for *have-to* and *want-to* goals in their paper, Milkman et al. [12] provided clear manipulations for *want*/*should* model in empirical research. Therefore, we use the *want*/*should* model to assess behavior when individuals face a variety of choices. We propose that how individuals balance between *want*/*should* behaviors can be explained by the ego depletion theory.

### Ego depletion and self-control

The ego depletion theory has been frequently cited over the past in self-control research in most fields of psychology, thus we adopt it to explain the effects of mindset switching. The ego depletion theory is widely cited in organizational behavior (e.g., [24, 25]) and consumer behavior (e.g., [26–28]). The basic concept of ego depletion is that our volitional behavior draws on a limited resource pool, akin to one’s physical strength, and that exertion of any executive cognitive function temporarily depletes our self-control resources. The exhaustion of self-control resources is termed *ego depletion*. Any behavior that requires an executive function, such as initiating and inhibition behavior [29], comparing different options [30], and tackling uncertainty [31], depletes self-control resources. Because of our limited resource pool, an act of cognitive exertion will have a detrimental effect on the following volition because it reduces our self-control resources [10, 11, 29, 32]. Therefore, ego depletion explains why exertion in one stage leads to loss of self-control in the subsequent stage.

As noted earlier, mindset switching, which depletes our self-control resources, occurs when we shift from one mode of thinking to another mode. Individuals can switch mindsets and mental tasks, but doing so requires resources and comes at a cognitive cost [4, 7]. Mindset switching involves disengaging from one mindset and then actively engaging a different, possibly opposing, mindset. It is an act of initiating and inhibition, a volitional behavior that requires self-control resources. The ego depletion theory claims that diverse acts of volition deplete a limited resource pool [29]. Therefore, each instance of mindset switching consumes self-control resources, thereby reducing them for subsequent self-control behavior. In other words, both the external cues, such as the demands of a task, and the internal cues, such as one’s attention pulling to other interesting ideas, like mind wandering can prompt individual switch mindsets. Interruptions at work are external cues that demand an individual to switch mindsets. In experiments on mindset switching, Hamilton et al. used sequential priming tasks (e.g., language switching) to stimulate participants to switch mindsets, and found evidence that mindset switching taxed self-control resources and impeded subsequent self-control tasks [3].

Balancing between the *want* and *should* self is influenced by the sufficiency of the decision maker’s self-control resources. People must exert effort and monitor their progress to achieve *should* goals. Examples of *should* behavior include choosing healthy foods, studying hard, doing physical exercise, and quitting smoking [12, 21, 22]. In contrast, the *want* self is associated with short-term pleasures. *Want* tasks require less self-control resources to perform than *should* tasks. Examples of *want* behavior include eating tasty desserts, buying self-indulgent products, and taking addictive drugs [12, 21, 22]. Therefore, people require self-control resources to consciously monitor and align their behavior with their *should* goals, and to inhibit their behavior from pursuing *want* goals.

Based on the preceding discussion, we posit that sufficient self-control resources increase our tendency to engage in *should* behaviors, whereas insufficient self-control resources increase our tendency to engage in *want* behaviors. Since mindset switching depletes self-control resources, we propose the following hypotheses.

*Hypothesis 1*: *Mindset switching influences individuals’ subsequent decisions*, *such that they will prefer “want” behaviors over “should” behaviors*.
*Hypothesis 2*: *The relationship between mindset switching and the subsequent choice of “want/should” behaviors is mediated by individuals’ state of ego depletion*.

This study contributes to the literature by linking mindset switching to *want*/*should* behavior and examining the mediating role of ego depletion. Researchers have demonstrated that mindset switching is an executive function that has negative effects on subsequent self-control tasks [3]. However, because of frequent mindset switching and the diverse behaviors required in social and work life, individuals choose different behaviors *before* engaging in a self-control task. Thus, the question of how mindset switching influences *want/should* behavioral choices is worth investigating. Moreover, this research makes a theoretical contribution by linking ego depletion theory to the multi-selves model. Specifically, we provide a theoretical explanation for the consequences of mindset switching. By doing so, this research also provides practical suggestions for improving individuals’ executive functions in today’s organizations where mindset switching is often required.

We now clarify how we manipulate *want/should* behavior in this study. The distinction between *want* behavior and *should* behavior is not always clear [23]. Some *should* behaviors (e.g., writing, jogging) may be enjoyable for those who like these behaviors, as these *should* behaviors may also be perceived (by those who like them) as *want* behaviors. Some *should* behaviors (e.g., pursuing career dreams, losing weight, saving money) have attractive outcomes and are quite appealing and can be considered *want* behaviors (by those who like engaging in them). Researchers have explained that the motivation behind behavior is a critical standard to define *want/should* behaviors [23, 33]. In this study, because the *want*/*should* behavior is the dependent variable and its underlying mechanism is not our research concern, we use Milkman et al.’s definition of *want/should* behaviors [12]: *want* behaviors are behaviors that bring primarily instantaneous pleasure, and *should* behaviors as those that bring primarily long-term utility. With this definition, all of the behaviors mentioned in this paragraph can be classified as *should* behaviors. In our study, we use confirmation questions (i.e., whether this behaviors bring you long-term utility) to ensure that all of our participants correctly understood our definition.

Skepticism also surrounds the concept of self-control resources, as no study has directly observed them, to the best of our knowledge. Instead, most researchers have inferred the ego depletion effect based on volitional behavior change between two sequential self-control tasks [25, 27, 29]. Although Gailliot has demonstrated that blood glucose levels are directly connected to self-control resources [34], whether the effects of ego depletion may be alternatively explained by short-term fatigue or motivational reasons requires investigation. Thus, it is valuable to eliminate alternative explanations of fatigue and motivation, which is what our study sets out to do.

## Ethical statement

The research ethics committee of the Institute of Human Resource Management, Zhejiang University approved the protocol of the following four experiments, including the purpose, procedure, and materials. The subjects provided written consent before taking part in this experiment and were fully debriefed on the purpose of the study afterwards. All participants were informed that all data (but not their specific individual responses) would be statistically analyzed at the group level, and that the data would be used only for academic purposes.

## Experiment 1: Effect of individualist/collectivist mindset switching on reading choices

Experiment 1 examines the main effect of how mindset switching influences the subject’s choice of *want*/*should* behavior. We used the individualist and collectivist mindsets to manipulate mindset switching. The individualist mindset focuses on self-interest and autonomy, values personal characteristics, and distinguishes the self from others. In contrast, the collectivist mindset focuses on group interactions and interpersonal connections, values the relationships between group members, and defines self by the recognition of others. Individualist and collectivist mindsets are formed by long-term participation in specific culture, but also can be temporarily changed by situational priming [35]. In empirical studies, researchers have used individualist and collectivist priming questions successfully to manipulate mindset switching [3]. Therefore, we used individualist and collectivist mindset switching to test Hypothesis 1.

### Methods

#### Participants

Experiment 1 was conducted at a large university located in eastern China. Ninety-one Chinese university students (63 females and 28 males, *M*_*age*_ = 20.88, *SD* = 2.01) were recruited. They were compensated CNY15 (approximately US$2.50) for their participation.

#### Procedures

When the participants arrived at the laboratory, they were randomly assigned to one of three conditions in the waiting room: individualist, collectivist, or mindset switching (individualist/collectivist). In the first stage for all three groups, the participants were asked to answer 9 open questions in a cubicle on computer. Drawing on past research [3], we asked each participant to write two statements for each question (See S1 Table). The statements for the individualist mindset group emphasized the self (e.g., Write two statements describing yourself.), the statements given to the collectivist mindset group focused on interpersonal perspectives (e.g., Write two statements describing groups to which you belong.), and both types of questions were presented alternately and randomly to the third group, the mindset-switching group. Immediately after finishing the answers, the participants evaluated their perceived effort and task difficulty (aimed to measure their individual ego depletion) [29]. After the experiment, as a manipulation check, a judge who was blind to the conditions was asked to review the participants’ statements as to whether the statements answered the question prompts [3].

In the second stage, all participants were instructed to choose reading materials for the following 20 minutes. The instructions were as follows: “Please choose between scientific journals and popular magazines to read. You will have the next 20 minutes to read what you have chosen.” The instructions also noted the properties of the two choices: “The scientific journals will enable you to enhance your knowledge, but will require more effort and concentration. In contrast, reading popular magazines is relaxing, but meaningless in terms of learning. Please choose your readings and assess your willingness for both types of journals.” Subsequently, the participants expressed their willingness to read a particular type of material on a 7-point Likert scale (1 = not at all, to 7 = very much), and then each participant completed the Positive and Negative Affect Schedule (PANAS) as a control variable [36]. To convince the participants to believe that they would read the magazines they selected, we had pre-arranged 20 different Chinese journals (ranging from scientific journals, e.g., *Management World*, *Psychological Science*, to popular magazines, e.g., *Stories*, *Comic Monthly*.) in the waiting room. They participants were informed generally that the journals would be used in the experiment.

Finally, to confirm that the participants understood *want*/*should* behavior correctly, each was asked to read the definition of *want*/*should* behavior and rated the scientific journal and popular magazines with the confirmation question. They evaluated the extent to which reading scientific journals and popular magazines could be categorized as *want* or *should* behavior according to the definition provided on a 7-point Likert scale (1 = “definitely a *want* behavior” to 7 = “definitely a *should* behavior”). After they finished their work in the lab, the participants were guided back to the waiting room again. The experimenter informed them that the choice of readings was a hypothetical scenario and that the experiment was over.

### Results

The comparison of the confirmation questions showed that all of the participants correctly understood the experiment’s definition of *want*/*should* behavior. The participants significantly categorized the popular magazines (*M* = 2.01, *SD* = 1.13) as more *want*-oriented than the scientific journals (*M* = 6.44, *SD* = 0.83), *t*(90) = 27.77, *p* < 0.001. On the individual level, all participants evaluated the popular magazines as being more *want*-oriented than the scientific journals.

For the mindset-switching manipulation check, the blinded conditional judge evaluated the participants’ answers. As expected, all of the sentences were written in the individualist or collectivist statements (e.g., the sentences beginning with “I am” reflected mindsets of the individualist group, the sentences beginning with “We are” reflected the collectivist group. The participants in the mindset-switching group wrote both types of sentences alternatively following the instructions.

The results for scientific journal/popular magazine-reading choice support Hypothesis 1. A chi-square test shows that mindset switching had a significant effect on *want*/*should* reading choices, *χ*^*2*^(1, N = 91) = 4.44, *p* = 0.035. The percentage of those choosing popular magazines in the non-switching group (we combined the individualist and collectivist groups into one non-switching group here) is 60.00%, but it rises to 80.49% in the mindset-switching group. The ANOVA results show that the mindset-switching manipulation had a significant effect on the ego depletion measures (α = 0.68), *F*(2, 88) = 4.21, *p* = 0.018, η^2^ = 0.09. The average effort and task difficulty for the mindset-switching group (*M* = 4.52, *SD* = 1.27) is significantly greater than that of the non-switching group (*M* = 3.77, *SD* = 1.23, *p* = 0.005). Post hoc comparisons using the Tukey HSD test indicate that the individualist group (*M* = 3.86, *SD* = 1.21) and the collectivist group (*M* = 3.67, *SD* = 1.28) have no significant difference (*p* = 0.842).

A comparison of the results of the willingness to choose the *want*/*should* reading selection also supports Hypothesis 1. The effect of mindset switching on the intention to read popular magazines is significant, *F*(2, 88) = 3.82, *p* = 0.026, η^2^ = 0.08. The intention of the mindset-switching group (*M* = 5.49, *SD* = 1.31) is significantly greater than that of the overall non-switching group (*M* = 4.74, *SD* = 1.63, *p* = 0.019). Tukey HSD comparisons indicate that the individualist group (*M* = 5.04, *SD* = 1.49) and the collectivist group (*M* = 4.46, *SD* = 1.73) have no significant difference (*p* = 0.355). Although the effect of mindset switching on the intention to read scientific journals is consistent with the prediction, it is not statistically significant, *F*(2, 88) = 2.05, *p* = 0.135, η^2^ = 0.04.

When individuals face a large number of choices, their decisions are influenced by their comparison of multiple options. To assess the comparison of *want*/*should* behavior, researchers developed the *Should-minus-Want* (*SMW*) scores for supermarket goods [21] and *Quickflix* (rental) movies [22]. We calculated *SMW* scores using a similar method based on the participants’ intentions to perform *should* behaviors (i.e., read scientific journals) minus their intentions to carry out *want* behaviors (i.e., read popular magazines). The effect of mindset switching on the *SMW* score is significant: *F*(2, 88) = 4.53, *p* = 0.013, η^2^ = 0.09. The *SMW* score of the mindset-switching group (*M* = -1.59, *SD* = 2.60) was significantly lower than that of the overall non-mindset switching group (*M* = -0.28, *SD* = 2.38, *p* = 0.014). Tukey HSD comparisons showed that the individualist group (*M* = -0.87, *SD* = 2.33) and the collectivist group (*M* = 0.27, *SD* = 2.34) had no significant difference (*p* = 0.233).

We argue that mindset-switching requires mental effort (i.e., it depletes ego strength) and thus influences participants to choose *want* over *should* options (Hypothesis 1) and that ego depletion mediates this relationship (Hypothesis 2). To test our hypotheses, we followed Hayes and Preacher’s [37] approach to test the mediation of multi-categorical independent variables. First, we created two dummy variables with the indicator coding [37]. In Experiment 1, we specified *Dummy1* of the switching condition (indicator coded as switching group = 1, collectivistic group = 0, individualistic group = 0) as the independent variable, and *Dummy2* (indicator coded as switching group = 0, collectivistic group = 1, individualistic group = 0) as a covariate. The indicator coding is listed in Table 1. In all of our experiments, we specified *Dummy1* as the switching condition and also the independent variable, and *Dummy2* as a covariate.

**Table 1:** Indicator coding in Experiment 1, 2, 3, 4.

*Dummy1*	*Dummy2*	Experiment 1	Experiment 2	Experiment 3 &4
1	0	Switching	Switching	Switching
0	1	Collectivistic	How	Chinese
0	0	Individualistic	Why	English

**Fig 1:**
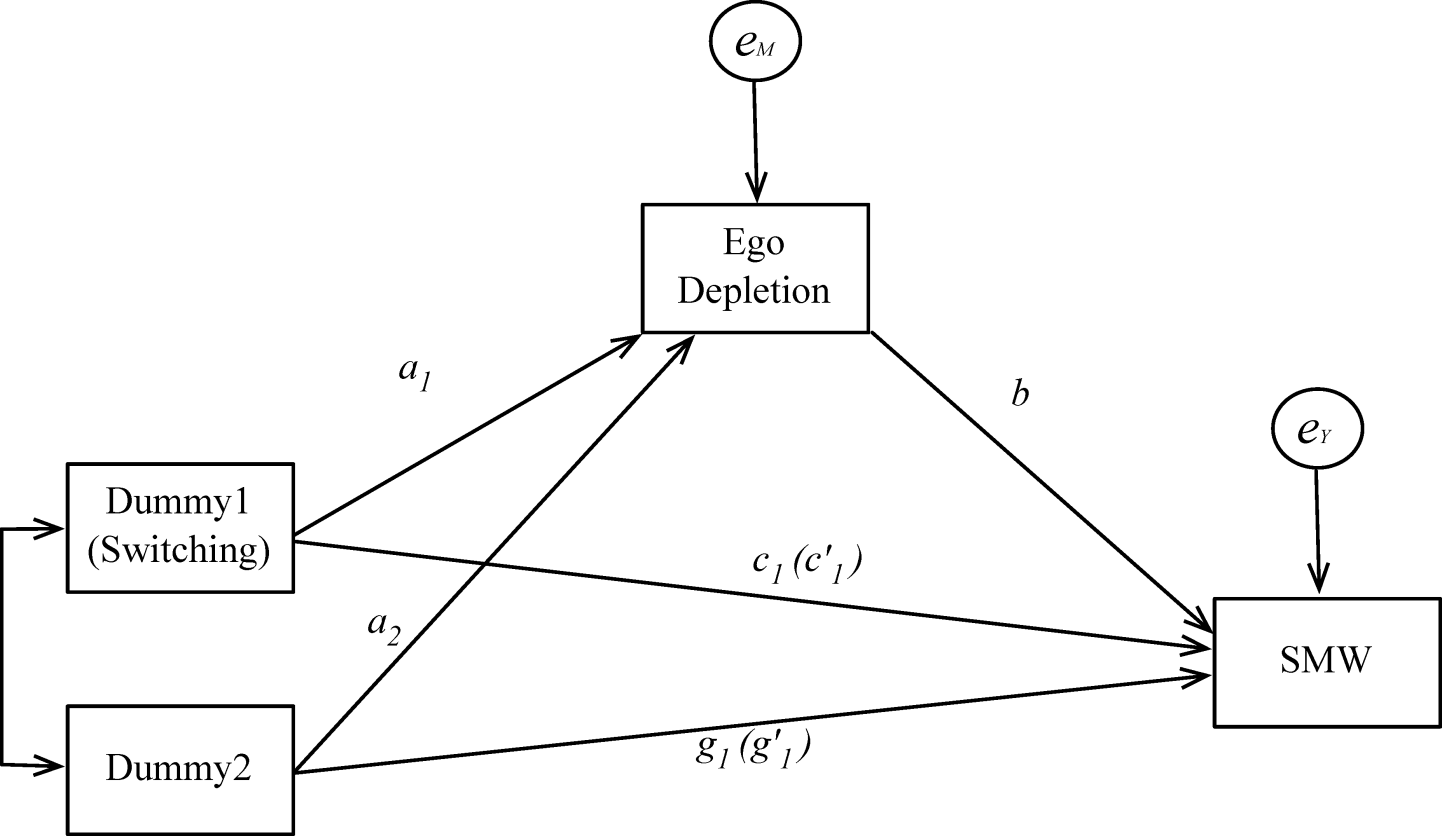
The PROCESS model as tested in all four experiments.

We tested our hypotheses using Model 4 illustrated in Fig 1 (with 10,000 bootstrapping samples and a confidence interval of 95%) of PROCESS models [37]. We used ego depletion as a mediator variable and demographic variables (age and gender) as control variables (See Table 2).

**Table 2:** Estimated coefficients by PROCESS in Experiment 1.

	M (Ego Depletion)			Y (SMW)					
		Coeff.	*SE*		Coeff.	*SE*		Coeff.	*SE*
**X** (Dummy1)	*a*_*1*_	0.84*	0.33	*c*_*1*_	-1.04^#^	0.59	*c'*_*1*_	-1.33*	0.60
**M** (Ego Depletion)							*b*	0.35^#^	0.19
**C**_**1**_ (Dummy2)	*f*_*1*_	0.15	0.36	*g*_*1*_	0.94	0.65	*g'*_*1*_	0.86	0.64
**C**_**2**_ (Age)	*f*_*2*_	-0.03	0.07	*g*_*2*_	0.00	0.11	*g'*_*2*_	0.01	0.12
**C**_**3**_ (Gender)	*f*_*3*_	-0.13	0.29	*g*_*3*_	-2.10**	0.52	*g'*_*3*_	-2.05**	0.52
**Constant**	*i*_*1*_	4.58**	1.52	*i*_*2*_	-2.88	2.73	*i*_*3*_	1.29	2.84
*R*^*2*^	0.09^#^			0.24**			0.27**		
*F*	2.17			6.67			6.14		

^#^p < .10

*p < .05

**p < .01

As expected, mindset switching had a direct effect on the *SMW* score, and the 95% confidence interval did not include zero, [-2.54, -0.13]. But the indirect effect of ego depletion was marginally significant, as the 95% confidence interval included zero, [-0.02, 0.88], but the 90% confidence interval did not include zero, [0.03, 0.78]. These results supported Hypothesis 1 that the participants in the mindset-switching group would prefer popular magazines to scientific journals more than those in the non-switching group, which suggests that switching between individualist and collectivist mindsets can prompt the desire for *want* behavior. Thus, Hypothesis 2 is partially supported by the marginally significant indirect effect.

The PANAS score comparisons eliminated the influence of emotion, such that the mindset-switching manipulation did not impact the positive emotions (e.g., interested, excited), *F*(2, 88) = 0.66, *p* = 0.519, η^2^ = 0.02, or negative emotions (e.g., distressed, irritable), *F*(2, 88) = 0.14, *p* = 0.869, η^2^ = 0.00.

### Discussion

The results of Experiment 1 support Hypothesis 1: switching between individualist and collectivist mindsets triggers a tendency for *want* behavior. In other words, mindset switching promotes self-indulgence.

To improve the validity of our conclusion, we will implement an alternative method to measure and manipulate variables. Researchers have adopted various *want*/*should* behavior manipulations [21, 22], and we followed the systematic approach developed by Milkman et al. [22] to test the reliability and validity of the *want*/*should* behavior measurement. Furthermore, to boost the validity of the robustness of our conclusion, we used an alternative method to manipulate mindset switching. The subsequent experiments aim to enhance the validity of the findings by using alternative manipulations.

Additionally, we speculate that the relative low reliability of ego depletion measures in Experiment 1 might be why its mediating effect has not been fully supported. The two-item measure of ego depletion has been adopted as a manipulation check of ego depletion [29], but seldom used as a measure in itself. In the following three experiments, we use an alternative ego-depletion scale and mindset switching manipulations to test the robustness of our two hypotheses.

## Experiment 2: Effect of abstract/concrete mindset switching on online behavior

In Experiment 2, we used an abstract/concrete mindset switching exercise to test both the main effect and mediating effect of ego depletion on our participants.

We used an abstract/concrete switching exercise to manipulate the independent variable, mindset switching. Construal level theory [38] proposes that individuals represent social events, actions, and goals on either the abstract or concrete level. Construal level means how individuals perceive, comprehend, and interpret the world such as abstract versus concrete, schematic versus aschematic, and prototype versus exemplar, within the umbrella of high- versus low-level construal. High-level construal is an abstract mental representation that is congruent with long-term goals, and low-level construal is a concrete mental representation that contains local constraints [39]. Researchers can activate participants’ abstract mindset by asking “why” questions and stimulate their concrete mindset by asking “how” questions [16]. Thus, in this experiment, we adopted an abstract/concrete mindset switching exercise to test Hypotheses 1 and 2.

### Pretest

In the pretest, we developed a *want*/*should* scale for online behavior. Ravizza et al. found that nonacademic Internet use was common among students who used laptops in university classrooms and was inversely related to academic performance [2]. Online behavior is an appropriate domain in which to measure the *want*/*should* conflicts of college students, since they spend a significant amount of time online and their online behavior includes both *should* behavior (e.g., searching for academic papers, reading articles) and *want* behavior (e.g., playing Internet games, emailing friends) in class. We referred to the *SMW* scoring steps developed by Milkman et al. [22] to create a *want*/*should* online behavior scale.

First, we recruited 10 university students (6 females, 4 males) for a semi-structured interview to answer the following questions: (1) “What do you usually do when using the Internet?”, (2) “What forms of online behavior do you think are beneficial to your studies?”, and (3) “What forms of online behavior do you think are unrelated to your studies?” Two research assistants coded online behavior, and then placed them in one of two categories: study-promoting (10 items) and entertaining (9 items).

Second, we recruited 87 participants (56 females and 31 males, *Mage* = 23.01, *SD* = 1.88) to evaluate the extent of *want*/*should* online behavior. The participants read the definition of *want*/*should* behavior by Milkman et al. [12] and then evaluated the extent to which each of the 19 items could be categorized as *want* or *should* behavior using a 7-point Likert scale (1 = “not at all this type of online behavior” to 7 = “definitely this type of online behavior”). The *SMW* score for each item was then calculated by subtracting the *wants* from the *shoulds* score. We also calculated the average and standard deviation of the *SMW* score for each item. Ten items (five items for *want* and five items for *should* online behavior) with very high or very low averages and relatively small standard deviations were selected as the scale items (S2 Table).

### Methods

#### Participants

Experiment 2 was conducted at a large university located in eastern China. We recruited 139 participants (70 female and 69 male, *M*_*age*_ = 20.88, *SD* = 2.16) through a university online Bulletin Board System. They were compensated CNY15 (approximately US$2.50) for participating in the experiment.

#### Procedures

First, the participants were randomly assigned to the priming tasks, which require them to answer questions beginning with either “why” or “how”. The why/how questions elicited the participants’ abstract/concrete mindsets [16]. We required the participants to answer seven “why” or “how” questions about their daily behavior in one sentence (e.g., “Why [or How] do you exercise?” and “Why [or How] do you travel?”). The participants were randomly assigned to one of three conditions: (1) answered only *why* questions (abstract mindset group), (2) answered only *how* questions (concrete mindset group), or (3) answered both types; switched between *why* and *how* questions (the mindset-switching group). After finishing the experiment, as a manipulation check, a research assistant who was blind to the manipulated conditions reviewed whether the respondents correctly followed the why/how directions, and found they all complied. Following this, effort and task difficulty questions were given to the participants as measures of ego depletion after the manipulation [29]. After this, using a 9-point Likert scale, the participants evaluated their intentions to engage in the actions listed in the *want*/*should* online behavior (1 = “very low” to 9 = “very high”).

#### Results

The ANOVA results showed that the mindset-switching exercise had a significant effect on the two-item measure of difficulty and effort (α = 0.67), *F*(2, 136) = 4.98, p = 0.008, η^2^ = 0.07. The reliability of the ego depletion measure was similar to that in Experiment 1; this result suggests that higher reliability measures should be adopted in the following experiments. The average for the ego depletion items for the mindset-switching group (*M* = 5.22, *SD* = 1.33) was significantly higher than that of the two non-switching conditions (*M* = 4.50, *SD* = 1.34, *p* = 0.002). Post hoc comparisons using the Tukey HSD test indicated that the concrete mindset group (*M* = 4.58, *SD* = 1.58) and the abstract mindset group (*M* = 4.43, *SD* = 1.08) had no significant difference in scores (*p* = 0.874).

The ANOVA results also demonstrated that mindset switching had a significant effect not only on *should* online behavior, *F*(2, 136) = 8.32, *p* < 0.001, η^2^ = 0.10, but also on *want* online behavior, *F*(2, 136) = 3.54, *p* = 0.032, η^2^ = 0.05. The *should* intention of the mindset-switching group (*M* = 2.53, *SD* = 1.33) was significantly lower than that of the overall non-switching group (*M* = 3.48, *SD* = 1.74, *p* = 0.001). Post hoc comparisons using Tukey HSD indicated that the abstract mindset group (*M* = 3.14, *SD* = 1.35) and the concrete mindset group (*M* = 3.84, *SD* = 2.03) had no significant difference (*p* = 0.109). The *want* intention of the mindset-switching group (*M* = 4.98, *SD* = 1.72) was significantly higher than that of the two non-switching group (*M* = 4.31, *SD* = 1.74, *p* = 0.011). The Tukey HSD comparison showed that the abstract mindset group (*M* = 4.20, *SD* = 1.38) and the concrete mindset group (*M* = 4.42, *SD* = 1.30) had no significant difference (*p* = 0.778).

We calculated the *SMW* scores with the participants’ average score on *want* online behavior minus their average score on *should* online behavior. A comparison of the *SMW* scores was significant, *F*(2, 136) = 9.45, *p* < 0.001, η^2^ = 0.12. The *SMW* score of the mindset-switching group (*M* = -2.45, *SD* = 1.84) was significantly lower than that of the overall non-switching group (*M* = -0.82, *SD* = 2.45, *p* < 0.001). A Tukey HSD comparison showed that the abstract mindset group (*M* = -1.06, *SD* = 1.91) and the concrete mindset group (*M* = -0.59, *SD* = 2.91) had no significant difference (*p* = 0.596). The results of the group mean comparisons support Hypothesis 1 that mindset switching had a negative effect on *SMW* scores. This suggests that the participants favored *want* options over *should* options to a greater extent after mindset switching than without mindset switching.

To test the mediating role of ego depletion, we also followed Hayes and Preacher’s [37] approach to test the mediation of multi-category independent variables. Like to results shown in Table 1, we specified the *Dummy1* of the switching condition (indicator coded as a switching group = 1, concrete mindset group = 0, abstract mindset group = 0) as the independent variable, and the *Dummy2* (indicator coded as switching group = 0, concrete mindset group = 1, and abstract mindset group = 0) as a covariate. We also tested the model in Fig 1 using the PROCESS Model 4 (with 10,000 bootstrapping samples and a confidence interval of 95%) [37]. The estimated coefficients are listed in Table 3.

**Table 3:** Estimated coefficients by PROCESS in Experiment 2.

	M (Ego Depletion)			Y (SMW)					
		Coeff.	*SE*		Coeff.	*SE*		Coeff.	*SE*
**X** (Dummy1)	*a*_*1*_	0.82**	0.28	*c*_*1*_	-1.40**	0.46	*c'*_*1*_	-1.01*	0.46
**M** (Ego Depletion)							*b*	0.46**	0.14
**C**_**1**_ (Dummy2)	*f*_*1*_	0.15	0.30	*g*_*1*_	0.46	0.50	*g'*_*1*_	0.53	0.48
**C**_**2**_ (Age)	*f*_*2*_	-0.04	0.05	*g*_*2*_	0.00	0.09	*g'*_*2*_	-0.01	0.09
**C**_**3**_ (Gender)	*f*_*3*_	0.13	0.23	*g*_*3*_	0.07	0.38	*g'*_*3*_	0.07	0.37
**Constant**	*i*_*1*_	5.19**	1.12	*i*_*2*_	-1.15	1.87	*i*_*3*_	1.25	1.94
*R*^*2*^	0.07*			0.12**			0.19**		
*F*	2.58			4.66			6.24		

*p < .05

**p < .01

The standardized indirect effect (path *a*_*1*_*b*) was -0.38, and the 95% confidence interval was [-0.77, -0.13]. The total effect of *Dummy1* on *SMW* was -1.40, and the 95% confidence interval was [-2.31, -0.48]. None confidence intervals included zero, therefore, both effects are statistically significant. Thus, the PROCESS results support both of our hypotheses that mindset switching influenced the participants’ *want/should* online behaviors via ego depletion.

### Discussion

The results of Experiment 2 strengthened our conclusion. We developed a *want*/*should* behavior scale using Milkman et al.’s procedure [22], which enhanced the constructive validity. We also employed an alternative mindset-switching manipulation that strengthened the internal validity of our conclusion. The results support Hypothesis 1 and Hypothesis 2. In other words, the results indicate that the relationship between mindset switching and *want*/*should* behavior can be mediated by self-control resource sufficiency.

In Experiment 3, we aimed to eliminate possible alternative explanations, such as fatigue and motivation, for the participants’ *want/should* choices. Fatigue plays a central role in behavior, particularly behavioral choices. In both Experiments 1 and 2, the dependent measurements of the *should* self were closely connected to the participant’s cognitive capacities. A possible alternative explanation is that mindset switching may influence cognitive fatigue, and may affect the individual’s subsequent behavioral choices. The overlap between ego depletion and fatigue is often discussed in the literatures [40]. Although evidence has shown some distinctions, for example, ego depletion increases aggression but fatigue decreases aggression, its effects have not been clearly distinguished. Thus, we set out to clarify whether cognitive fatigue influences *want/should* choices in addition to ego depletion.

Another possible alternative explanation is motivational differences. Researchers have argued that motivation plays an important role in ego depletion [23, 33]. A high level of motivation may give people unlimited resources in some tasks, and may also moderate ego depletion effects [11]. The Need for Cognition is a dispositional difference that reflects the extent to which individuals are inclined to participate in cognitive activities [41]. In other words, individuals with a high Need for Cognition prefer cognitive tasks, such as reading and thinking. Therefore, Need for Cognition can be considered a motivational difference in *want*/*should* reading choices. In Experiment 3, we examine how the motivational difference might affect *want/should* choices.

## Experiment 3: Effect of Chinese/English language switching on reading choices

We used language switching as a mindset-switching task in Experiment 3 with native Chinese-speaking participants because language can serve as a priming condition (a manipulation) to shift individuals’ cultural mindsets. Cultural mindset is a cognitive schema containing culture-congruent content, procedures, and goals, such as individualism and collectivism [17]. The culture-specific mental frames or mindsets of bilingual individuals are activated when they speak a particular language [42]. For the past 20 years, English has been taught in China’s middle schools, high schools and universities, resulting from China’s open-door policies and education reforms of the 1990s. Although our participants’ native language was Chinese (e.g., Mandarin, Cantonese, Hunanese), they had studied English for over 10 years and could use it in their daily lives. They could fluently understand, read, and write short essays in English. Moreover, the Western cultural mindset had taken root during 10 years of English study. Following the Chinese and English cultural mindsets of bilingual (Chinese-English) study participants was activated when speaking Chinese and English respectively [43]. Therefore, we adopted questions to elicit the participants to think in Chinese/English in Experiment 3.

The experiment’s objective was to replicate the results of our previous two experiments, and to eliminate the alternative explanations of fatigue and motivation. We examined the effect of Chinese/English mindset switching on their *want*/*should* reading choices, as mediated by the level of ego depletion. We also examined the influence of Need for Cognition and cognitive fatigue.

### Methods

#### Participants

Experiment 3 was conducted at a top university located in eastern China. Participants included 107 undergraduate native-Chinese-speaking students (87 females and 20 males, *M*_*age*_ = 20.68, *SD* = 2.10). They were compensated CNY15 (approximately US$2.50) for participating in the experiment.

#### Procedures

irst, the participants completed the 18-item Need for Cognition Scale [44] using the adapted Chinese version [45] to measure each individual’s Need for Cognition as a motivational factor of their reading choices of either the scientific journal or popular magazine category. After a 2-minute break following completion of the Need for Cognition Scale, the participants were randomly assigned to one of three conditions: Chinese language, English language, or switching between both languages, to answer 18 open-ended questions about their social lives (e.g., “Please describe how you interact with your classmates in Chinese (Chinese condition)”, “Please describe the characteristic that distinguishes you from your classmates in English (English condition)”. Chinese and English are required alternatively and randomly in the switching group). It took approximately 20 minutes for the participants to complete the language switching priming task.

As a manipulation check, a research assistant who was blind to the switching conditions reviewed all of the participants’ responses as to whether they had answered the questions in the designated language. In this experiment, we updated the two items of the ego depletion measure from Milkman [31]. The participants were asked on a Likert scale about “the extent to which they felt too tired to select the scientific journals” and “the extent to which they felt too worn out to select the scientific journals” (1 = “not at all” to 7 = “very much”).

After the language-switching task, the participants then were directed to select their reading materials (i.e., scientific journals or popular magazines) and to evaluate their intentions to read them on a 7-point Likert scale (1 = “not at all” to 7 = “very much”). As what we do in Experiment 1, we had pre-arranged 20 Chinese journals in the waiting room to prompt the participants to believe that they will really read what they choose. Then, we gave the participants a 7-point Likert scale to complete in which they indicated the extent to which their choice to read scientific journals or popular magazines were *want* or *should* behaviors (1 = “could definitely be classified as a *want* behavior” to 7 = “could definitely classified as *should* behavior”).

Next, the participants were asked to complete a memory quiz to measure their short-term memory [46] and a Brief Mood Introspection Scale (BMIS) questionnaire to assess their emotional state [47]. This third experiment aimed to demonstrate that cognitive load influences the participants’ simple cognitive processing functions (e.g., short-term memory) and in contrast, that self-control resources influenced their higher-order functions of goal achievement (rather than simple cognition) [46]. Therefore, if mindset switching depletes self-control resources without increasing cognitive load, then it should affect their *want*/*should* choices, but not short-term memory performance.

### Results

All participants in our third experiment correctly understood the definition of *want*/*should* behavior based on our confirmation question. On the item level, they significantly categorized the popular magazines (*M* = 1.94, *SD* = 1.08) as more *want*-oriented than the scientific journals (*M* = 6.24, *SD* = 0.97), *t*(90) = 24.48, *p* < 0.001). On the individual level, all of the participants evaluated the popular magazines as being more *want*-oriented than the scientific journals.

A chi-square test demonstrated that language switching significantly influenced *want*/*should* behavior, *χ*^*2*^(1, N = 107) = 7.25, *p* = 0.007, supporting Hypothesis 1. In the Chinese-writing group, 77.8% of the 107 participants selected popular magazines. In the English writing group, 67.6% of the participants selected popular magazines. An average of 72.8% of the participants in the Chinese and English groups chose popular magazines, and the figure increased to 94.6% in the language-switching group.

Mindset switching had significant effects on ego depletion. We found a difference in ego depletion measures (α = 0.92) among the three groups, *F*(2, 104) = 4.41, *p* = 0.015, η^2^ = 0.08. The extent of ego depletion was significantly higher in the language-switching group (*M* = 5.53, *SD* = 1.11) than that of the two non-switching groups (*M* = 4.70, *SD* = 1.59, *p* = 0.006). The Tukey HSD test indicated that the Chinese-writing group (*M* = 4.54, *SD* = 1.77) and the English-writing group (*M* = 4.87, *SD* = 1.39) had no significant difference (*p* = 0.614).

The ANOVA also showed that mindset switching activity had a significant effect on the participants’ intention to read popular magazines, the *want* behavior, *F*(2, 104) = 4.43, *p* = 0.014, η^2^ = 0.08. The intention of the language-switching group (*M* = 6.00, *SD* = 0.82) was significantly higher than that of the two non-switching groups (*M* = 5.37, *SD* = 1.37, *p* = 0.012). Multiple comparisons using the Tukey HSD test showed that the Chinese-writing group (*M* = 5.58, *SD* = 1.23) and the English-writing group (*M* = 5.15, *SD* = 1.50) had no significant difference (*p* = 0.288). But the ANOVA results showed that language-switching had no significant effect on the intention to read scientific journals (the *should* behavior), *F*(2, 104) = 1.28, *p* = 0.282.

When the *SMW* score was the dependent variable, the ANOVA results demonstrated that the effect of the Chinese/English mindset switching was significant, *F*(2, 104) = 3.55, *p* = 0.032, η^2^ = 0.06. The *SMW* score of the switching group (*M* = -2.57, *SD* = 1.83) was significantly lower than that of the overall non-switching group (*M* = -1.43, *SD* = 2.52, *p* = 0.017). The Tukey HSD test indicated that the Chinese-writing group (*M* = -1.72, *SD* = 2.36) and the English-writing group (*M* = -1.12, *SD* = 2.68) had no significant difference (*p* = 0.519).

Following Hayes and Preacher [37], we also created two dummy variables with the indicator coding method. We specified the *Dummy1* of the switching condition (indicator coded as switching group = 1, Chinese group = 0, English group = 0) as the independent variable, and the *Dummy2* (indicator coded as switching group = 0, Chinese group = 1, English group = 0) as a covariate.

We tested our hypotheses with the PROCESS Model 4, shown in Fig 1, with 10,000 bootstrap samples and a confidence interval of 95%. The Need for Cognition Scale (α = 0.91) was included as a motivational independent variable, and the demographic variables (age and gender) were included as control variables (See Table 4).

**Table 4:** Estimated coefficients by PROCESS in Experiment 3.

	M (Ego Depletion)			Y (SMW)					
		Coeff.	*SE*		Coeff.	*SE*		Coeff.	*SE*
**X** (Dummy1)	*a*_*1*_	0.70*	0.34	*c*_*1*_	-1.47**	0.54	*c'*_*1*_	-0.84^#^	0.45
**M** (Ego Depletion)							*b*	0.90**	0.12
**C**_**1**_ (Dummy2)	*f*_*1*_	-0.27	0.35	*g*_*1*_	-0.66	0.54	*g'*_*1*_	0.89*	0.44
**C**_**2**_ (Age)	*f*_*2*_	-0.05	0.07	*g*_*2*_	-0.03	0.11	*g'*_*2*_	0.02	0.09
**C**_**3**_ (Gender)	*f*_*3*_	0.62^#^	0.36	*g*_*3*_	-0.72	0.56	*g'*_*3*_	-0.17	0.47
**C**_**4**_ (Need for Cognition)	*f*_*4*_	-0.02	0.22	*g*_*4*_	0.79*	0.35	*g'*_*4*_	0.77**	0.28
**Constant**	*i*_*1*_	2.69	1.63	*i*_*2*_	-1.77	2.56	*i*_*3*_	0.66	2.12
*R*^*2*^	0.11*			0.13*			0.42**		
*F*	2.47			2.99			11.96		

^#^p < .10

*p < .05

**p < .01

The results of the PROCESS coefficients showed that the standardized indirect effect (path *a*_*1*_*b*) was -0.63, and the 95% confidence interval was [-1.36, -0.14]. The total effect of *Dummy1* on *SMW* was -1.47, and the 95% confidence interval was [-2.53, -0.40]. No confidence intervals included zero, therefore, both effects were statistically significant. Thus, the PROCESS results supported both of our hypotheses.

Moreover, the PROCESS coefficients suggest that the Need for Cognition firmly predicted the participants’ preferences for *want*/*should* behaviors. Specifically, the participants with a high Need for Cognition expressed a preference for the scientific journals over popular magazines. After the Need for Cognition was statistically controlled in the model, the indirect effect (path *a*_*1*_*b*) of mindset switching on *SMW* remained significant. Thus, although the effect of Need for Cognition was controlled, nevertheless, both of our hypotheses were supported at .01 level.

In addition, as the ANOVA results show, as a measure of cognitive fatigue, short-term memory performance was not significantly influenced by mindset switching, *F*(2, 104) = 1.42, *p* = 0.246, η^2^ = 0.03. Thus, cognitive performance and *want*/*should* behaviors were not influenced synchronously after mindset switching. Furthermore, cognitive fatigue did not vary at the same pace as *want*/*should* behaviors. Language switching has no effect on emotions. Positive emotion (α = 0.86) and negative emotion (α = 0.80) scores were computed using the BMIS scale. The difference of the overall single-item BMIS score was not significant, *F*(2, 104) = 0.27, *p* = 0.761, η^2^ = 0.01. Neither positive emotions, *F*(2, 104) = 0.57, *p* = 0.568, η^2^ = 0.01, nor negative emotions, *F*(2, 104) = 2.08, *p* = 0.131, η^2^ = 0.04, was influenced by mindset switching.

### Discussion

Similar to the findings of the previous two experiments, language switching influenced the participants’ inclination for *want*/*should* behavior in Experiment 3. The regression results, once again, empirically supported the mediating role of ego depletion, thereby upholding Hypotheses 1 and 2. Furthermore, Experiment 3 strengthened this study’s internal validity by eliminating the two alternative explanations: cognitive fatigue and motivation.

Although the Need for Cognition explained part of the variance in our participants’ *want*/*should* behavior, ego depletion had stable effects on *want/should* choices even after the Need for Cognition was statistically controlled. Our multiple regression models showed that the Need for Cognition influenced reading choices, but the main effect of mindset switching and the mediating effect of ego depletion remained significant, the same as in our previous two experiments. Thus, Hypotheses 1 and 2 were reinforced by the PROCESS results with control of Need for Cognition.

The alternative explanation for cognitive fatigue was also eliminated. We adopted the short-term memory test as a measure of cognitive performance. If cognitive fatigue was an alternative explanation, mindset switching would have influenced both cognitive performance and ego depletion. However, our results showed that short-term memory performance was not impaired. This suggests that cognitive fatigue does not increase after mindset switching. It supports the hypothesis that mindset switching influences ego depletion but not cognitive fatigue. It also indicates that cognitive fatigue and ego depletion are two different cognitive concepts based on the evidence of Experiment 3.

A possible limitation of Experiments 1 to 3 is that all of the dependent variable measurements of *want*/*should* behaviors are hypothetical, but not actual behavior. Although in Experiment 2, we developed the online behavior scale using Milkman et al.’s method [22], the behavioral intentions that the participants evaluated were not actual online behavior. In Experiments 1 and 3, we used the choice between scientific journals and popular magazines as reading materials. Although we prepared the two types of magazines and prompted the participants to think that they would read the materials of their choice, we did not let the participants actually read what they chose. We set out to improve the validity of our findings with a fourth experiment, where we demonstrate our model with actual *want*/*should* behavior.

## Experiment 4: Effect of Chinese/English language switching on food choices

Experiment 4 aimed to test our two hypotheses with actual *want*/*should* behaviors, and to replicate the experimental results from our previous three experiments. We examined the effects of Chinese/English mindset switching on actual *want*/*should* food choices mediated by the participants’ ego depletion level.

The food choice task is a frequently cited measure of *want*/*should* behavior [31]. When individuals, especially those on a diet, choose foods to eat, most must balance their choices between tasty but unhealthy *want* foods and less tempting but healthy *should* foods. Most *want* foods, such as cookies, ice cream, candy, fried foods and fatty meats may be appetizing but are high in calories and low in nutrition, whereas most *should* foods, such as fruits, vegetables, and salads, are nutritious and low-calorie but much less appealing [21]. Therefore, in our sample who stated they were on a diet, we used an apple as the *should* food choice, and a package of M&M chocolate beans as the *want* food choice. Additionally, these foods were familiar to the participants and similarly priced in local supermarkets, so that price would not be a factor influencing the participants’ choices.

### Methods

#### Participants

Experiment 4 was conducted at a top university in eastern China. As we adopted actual food choices as the measures of *want*/*should* behavior, we emphasized in our recruiting advertisement on university BBS that we were seeking participants who were dieting. Furthermore, when the participants arrived at the laboratory, we asked each of them whether they were on a diet. The participants who answered affirmatively joined the experiment. 89 participants (79 females and 10 males, *M*_*age*_ = 20.61, *SD* = 1.64) were recruited. They were compensated CNY15 (approximately US$2.50) for participating in the experiment, plus they received the food they had chosen.

#### Procedures

The participants waited in the waiting room before they participate in Experiment 4. In this room, we had placed a tray of green apples and packages of M&M chocolates. We told them that these were “extra gifts” for their participation after this experiment. The participants were then guided to their cubic and told that they were participating in a language-practice task. They were randomly assigned to one of three conditions (i.e., Chinese, English, or English/Chinese switching), the same as in Experiment 3. As a manipulation check, a research assistant checked all of the participants’ responses as to whether they had answered in the designated language.

When the language-priming task was finished, the instructions on the computer screen encouraged the participants to take a snack before leaving the laboratory as a gift. They could choose between an apple and a small package of M&Ms [31]. Before they took the food, they were required to make their choice on the screen and evaluate their willingness to enjoy the food on a 7-point Likert scale (1 = “not at all” to 7 = “very much”). Similar to Experiment 1, the participants had already seen the apples and M&M chocolates in the waiting room. Pictures of an apple and a package of M&M chocolates were also displayed on the screen when they answered the questions about the foods. The participants were then asked to finish Milkman’s [31] two-item ego depletion scale (as in Experiment 3). Finally, the participants were asked to complete the BMIS questionnaire to assess their emotional state [47].

### Results

As a manipulation check, the judge confirmed that all of the participants had responded in the specified language (Chinese, English or switching between Chinese and English). Milkman’s [31] two-item ego depletion measure (α = 0.92) showed that language switching influenced their ego depletion levels significantly, *F*(2, 86) = 5.08, *p* = 0.008, η^2^ = 0.11. The average for the switching group (*M* = 5.04, *SD* = 1.47) was significantly higher than that of the overall non-switching group (*M* = 4.05, *SD* = 1.49, *p* = 0.003). Post hoc comparisons using the Tukey HSD test indicated that the Chinese group (*M* = 3.87, *SD* = 1.53) and English group (*M* = 4.23, *SD* = 1.45) had no significant difference (*p* = 0.640).

A chi-square test examined the effect of language switching on food choice. We combined the English- and Chinese-language groups into one non-switching group, and compared the results to those of the switching group. The effect of mindset switching was significant, *χ*^*2*^(1, N = 89) = 5.00, *p* = 0.042. The results of the actual *want*/*should* food choice behavior supported Hypothesis 1; that is, mindset switching influenced the subsequent choices of *want*/*should* foods in this experiment. In the non-switching groups, 51.79% of the participants selected the M&M chocolates as their snack. The ratio of M&Ms increased to 75.76% in the language-switching group.

The ANOVA results show that mindset switching significantly influenced the participants’ willingness to choose the apple, the *should* behavior, *F*(2, 86) = 5.00, *p* = 0.009, η^2^ = 0.10. The intention of the switching group (*M* = 4.00, *SD* = 1.92) was significantly lower than that of the non-switching groups (*M* = 5.16, *SD* = 1.62, *p* = 0.003). The Tukey HSD test indicates that the Chinese group (*M* = 5.36, *SD* = 1.57) and the English group (*M* = 4.96, *SD* = 1.67) had no significant difference on their choice of M&Ms, the *want* behavior (*p* = 0.675). Thus, we found that language switching had no significant effect on their intention to choose the M&Ms, the *want* behavior, *F*(2, 86) = 1.10, *p* = 0.337, η^2^ = 0.03. However, the trend in the means was consistent with Hypothesis 1. The *want* choice of the English/Chinese-language switching group (*M* = 6.06, *SD* = 1.07) was higher than that of the Chinese-language group (*M* = 5.68, *SD* = 1.36) and the English group (*M* = 5.64, *SD* = 1.25).

When the *SMW* score is the dependent variable, the ANOVA results show that the differences among the groups are significant, *F*(2, 86) = 5.96, *p* = 0.004, η^2^ = 0.12. The *SMW* score of the language switching group (*M* = -2.06, *SD* = 2.54) is significantly lower than that of the non-switching group (*M* = -0.50, *SD* = 2.41, *p* = 0.005). A Tukey HSD test indicates that the Chinese-language group (*M* = -0.32, *SD* = 2.34) and the English group (*M* = -0.68, *SD* = 2.51) had no significant difference (*p* = 0.852). Thus, the comparisons of the food selection intentions supported Hypothesis 1: the participants in the mindset switching group preferred *want* food over *should* food.

We also followed Hayes and Preacher’s [37] PROCESS approach to test Hypothesis 2. We used the same indicator coding system of *Dummy1* and *Dummy2* as in Experiment 3. The estimates are listed in Table 5.

**Table 5:** Estimated coefficients by PROCESS in Experiment 4.

	M (Ego Depletion)			Y (SMW)					
		Coeff.	*SE*		Coeff.	*SE*		Coeff.	*SE*
**X** (Dummy1)	*a*_*1*_	0.83*	0.38	*c*_*1*_	-1.34*	0.63	*c'*_*1*_	-0.83	0.60
**M** (Ego Depletion)							*b*	-0.62**	0.17
**C**_**1**_ (Dummy2)	*f*_*1*_	-0.27	0.40	*g*_*1*_	0.23	0.66	*g'*_*1*_	0.07	0.62
**C**_**2**_ (Age)	*f*_*2*_	0.02	0.09	*g*_*2*_	0.23	0.15	*g'*_*2*_	0.24	0.15
**C**_**3**_ (Gender)	*f*_*3*_	0.79	0.50	*g*_*3*_	-1.39^#^	0.83	*g'*_*3*_	-0.90	0.79
**Constant**	*i*_*1*_	2.31**	2.16	*i*_*2*_	-2.80	3.57	*i*_*3*_	-1.39	3.37
*R*^*2*^	0.13*			0.14 **			0.25**		
*F*	3.21			3.31			5.66		

^#^p < .10

*p < .05

**p < .01

The PROCESS coefficients show that the standardized indirect effect (path *a*_*1*_*b*) is -0.51, with a 95% confidence interval of [-1.15, -0.08]. The total effect of *Dummy1* on *SMW* is also -1.34, with a 95% confidence interval of [-2.59, -0.09]. No confidence intervals included zero, therefore, both the total and indirect effects were statistically significant. The PROCESS results supported both of our hypotheses. Thus, we replicated the mediating role of ego depletion observed in Experiment 3.

A comparison of the BMIS (emotion) scale results show that mindset switching does not have a significant effect on positive emotions (α = 0.84) nor on negative emotions (α = 0.80) in Experiment 4. The comparison of the overall single-item BMIS score was not significant, *F*(2, 86) = 0.09, *p* = 0.911, η^2^ = 0.00. Neither positive emotions, *F*(2, 86) = 0.05, *p* = 0.950, η^2^ = 0.00, nor negative emotions, *F*(2, 86) = 1.51, *p* = 0.227, η^2^ = 0.03, was influenced by mindset switching.

### Discussion

Experiment 4 provides empirical support for our two hypotheses using actual food choices. In our experiments to measure the participants’ intentions to perform *want*/*should* behaviors, we used reading choices (popular magazines and scientific journals) in Experiments 1 and 3, and hypothetical online behavior in Experiment 2. The results of the actual food choices in Experiment 4 provide additional direct evidence of *want*/*should* behavior. The replication of the mediating effects also strengthens the role that ego depletion plays in mindset switching. Thus, the results of Experiment 4 provide additional support for Hypotheses 1 and 2.

## General discussion

With these four experiments, we show that mindset switching increases individuals’ *want* behavior. This finding is robust across the various manipulations of mindset switching and various measures of *want/should* conflict. In addition, we demonstrate that ego-depletion is a vital mechanism that explains the effects of mindset switching on *want/should* behavior.

This study contributes to the literature first by providing evidence that mindset switching can induce individuals to prefer *want* behavior over *should* behavior. In other words, mindset switching not only has negative effects on cognitive performance (e.g., slower responses and higher error rates in a digit classification task [6, 7]), but lowers individuals’ ability to resist the temptation of immediate pleasure. Our results also demonstrate that ego depletion is the reason why mindset switching affects the choices of *want* or *should* behavior. Although behavioral researchers have found that motivation and cognitive fatigue might also explain the effect, our findings indicate that ego-depletion is the vital mechanism.

Secondly, this study contributes to literatures by linking ego depletion to *want*/*should* behaviors. Ego depletion impedes the subsequent executive functions aimed to achieve a clearly defined goal, such as persisting in handgrips and in completing impossible anagrams [48]. However, individuals might have different behavioral choices before they engage in goal-oriented behavior. The *want*/*should* behavior model suggests that we face internal conflicts when we make choices. This study provides evidence that ego depletion affect the internal conflicts when individuals make *want/should* choices in addition to influencing subsequent executive behavior.

A third contribution of our study is that our findings raise the question as to whether mindset switching can prompt unethical behavior. Similar to *want* behavior, unethical behavior is also tempting. It brings immediate benefits, but increases the long-term risks of sanctions (legal and social), and also increases threats to individuals’ self-concept. Research has shown that ego depletion increases the possibility of impulsive cheating [49], decreases the tendency for pro-social behavior [50], decreases moral awareness, and increases failure to resist temptation [25]. When we face tempting ethical situations (cheating on one’s taxes, or benefiting from a cashier’s error in a money exchange), we feel internal *want*/*should* conflicts, as we know we should resist short-term *want* benefits and choose the long-term *should* benefits (paying more taxes and giving back the extra change received in error so as to feel better about our moral or ethically correct behavior). Thus, researchers can test whether mindset switching can increase the risk of impulsive unethical behavior in organizations and in other social realms.

Finally, we provide some evidence that supports Inzlicht et al.’s [23] claim that various motivations can account for the apparent self-control failures. They assert that self-control failure can be avoided by creating an optimal balance between the cognitive labor of pursuing *have-to* goals and the cognitive leisure of pursuing *want-to* goals. In the literature on ego depletion, self-control hinges on the limited resource pool that enables us to pursue long-term utility versus short-term rewards. However, the results of Experiment 3 suggest that although the motivational factor of Need for Cognition predicts *want*/*should* choices, ego depletion cannot be excluded. Thus, our results show a significant influence of ego depletion on subsequent *want*/*should* behavior choices in addition to Need for Cognition.

This study’s findings also have practical implications for time management. Our study suggests that managers and employees should be aware of the consequences of mindset switching on *want/should* behavior. In order to motivate behavior that helps employees achieve the firm’s long-term goals, managers can try to reduce mindset switching by, for example, creating a work environment that blocks out frequent interruptions such as messages, phone calls, needless meetings. Brown provided a solution called the *Mutual Time Lock agreement* in which both the manager and employees are given a period of dedicated quiet time to concentrate [51]. Therefore, both parties can conserve their self-control resources and align their choice with *should* behaviors.

In addition, managers can intentionally avoid mindset switching to protect their limited self-control resources. For example, when writing an important report, and an e-mail pops up on the screen urging an immediate reply, the manager should avoid reading the email or any emails for that matter. Because, as outlined in the paper, if the manger decides to read the e-mail, he/she must switch mindsets from writing to answering the email, and then return to report writing, which depletes self-control resources and lowers motivation to write the report, *should* behaviors. We suggest that managers implement programs (e.g., software that blocks outside emails or internet surfing) to reduce employee mindset switching so as to preserve limited self-control resources and align work behavior with *should* behavior.

This study has some limitations that can be addressed in future studies. First, the effects of mindset switching on *want*/*should* behavior must be further explored. In this study, the *SMW* (*Should-minus-Want*) score was used as a compound measure. Although the results of the *SMW* scores correspond across the four experiments, mindset switching affected both *want* and *should* behaviors in Experiment 2, but only the *want* behaviors in Experiments 1 and 3, and only the *should* behaviors in Experiment 4. We suggest that the *SMW* score is a better indicator of *want/should* behaviors than a single item measure of *want* or *should*, as internal conflicts are only well described when individuals compare multiple options. *Want* and *should* behaviors are two sides of the same coin, and the *SMW* score provides more balanced information. Our correlation data results also show that the *SMW* score is more strongly correlated with *want*/*should* choices than either a single measure of *want* or *should*. Furthermore, the inconsistent results could be caused by the content of the options. Generally, the reading journals in Experiments 1 and 3, which required mental concentration, were closer to *should* options, whereas the selecting food in Experiment 4 was closer to *want* options. Thus, we encourage researchers to test the inconsistent effects of mindset switching on a single *want*/*should* measure.

Second, we did not directly measure self-control resources. As we employed ego depletion theory to explain the effects of mindset switching, the direct measure of self-control resources remains untested yet important. In recent decades, studies of self-control have been heavily influenced by the ego depletion theory, but the direct measure of self-control resources has remained unsolved. Several recent studies have proposed different models [23, 33] and have challenged the limited resource assumption of ego depletion [52]. Although we used the two-item scale that has been widely cited in ego depletion theory experiments, direct manipulations, such as sleep deprivation [53], and dynamic methods, such as experience sampling methods [54] could provide more comprehensive evidence.

## Supporting information

S1 Data Data for all experiments. (XLSX)

S1 Table Autobiographical writing tasks (mindset manipulation) in Experiment 1. (DOC)

S2 Table *Want*/*should* online behavior scale in study 2. (DOC)
