# Supplementary material for: Mindset switching increases the use of 'want-based' over 'should-based' behaviors
Source: PLoS One. 2018 Apr 30;13(4):e0196269. doi: 10.1371/journal.pone.0196269 (PMC5927408; doi:10.1371/journal.pone.0196269)
Supplement: S1 Table — (DOC) [file pone.0196269.s002.doc]

**Table S1. Autobiographical Writing Tasks (Mindset Manipulation) in Experiment 1**

| **Writing Task** | **Mindset Condition** | | |
| --- | --- | --- | --- |
|  | **Individualist** | **Collectivist** | **Switching** |
| 1. Write two statements describing | ...yourself. | ...groups to which you belong. | ...yourself. |
| 2. Write two statements about how you are | ...different from others. | ...like others. | ...like others. |
| 3. Write two sentences starting with | ...“I am.” | ...“We are.” | ...“I am.” |
| 4. Write two statements about the advantages of | ...standing out. | ...blending in. | ...blending in. |
| 5. Describe a gift you gave to | ...yourself. | ...someone else. | ...yourself. |
| 6. Write two statements describing | ...yourself. | ...groups to which you belong. | ...groups to which you belong. |
| 7. Write two statements about how you are | ...different from others. | ...like others. | ...different from others. |
| 8. Write two sentences starting with | ...“I am.” | ...“We are.” | ...“We are.” |
| 9. Write two statements about the advantages of | ...standing out. | ...blending in. | ...standing out. |

This table is cited from Hamilton et al. [36]
