# Supplementary material for: Mindset switching increases the use of 'want-based' over 'should-based' behaviors
Source: PLoS One. 2018 Apr 30;13(4):e0196269. doi: 10.1371/journal.pone.0196269 (PMC5927408; doi:10.1371/journal.pone.0196269)
Supplement: S2 Table — (DOC) [file pone.0196269.s003.doc]

**Table S2. *Want*/*should* online behavior scale in Study 2.**

***Want* online behavior**

1. Surfing Renren (*a website in China that is similar to Facebook)*
2. Surfing online shopping websites (e.g., Taobao)
3. Communicating with others via QQ or MSN
4. Surfing or posting statuses on Microblog
5. Surfing BBS (e.g., Tianya and Douban)

***Should* online behavior**

1. Replying work-related e-mails
2. Searching for academic papers in online libraries
3. Searching for and reading industrial reports
4. Using online databases
5. Sending online work-related notices
